# Crystal structure of 3-(thio­phen-2-yl)-5-*p*-tolyl-4,5-di­hydro-1*H*-pyrazole-1-carbo­thio­amide

**DOI:** 10.1107/S2056989015010774

**Published:** 2015-06-10

**Authors:** S. Naveen, G. Pavithra, Muneer Abdoh, K. Ajay Kumar, Ismail Warad, N. K. Lokanath

**Affiliations:** a Institution of Excellence, University of Mysore, Manasagangotri, Mysore 570 006, India; b Department of Chemistry, Yuvaraja’s College, University of Mysore, Mysore 570 006, India; c Department of Physics, Science College, An-Najah National University, PO Box 7, Nablus, Palestinian Territories; d Department of Chemistry, Science College, An-Najah National University, PO Box 7, Nablus, Palestinian Territories; e Department of Studies in Physics, University of Mysore, Manasagangotri, Mysore 570 006, India

**Keywords:** crystal structure, pyrazole, thio­phene, carbo­thio­amide, C—H⋯S hydrogen bonds, N—H⋯π inter­actions, π–π inter­actions

## Abstract

In the title compound, C_15_H_15_N_3_S_2_, the central pyrazole ring adopts a twisted conformation on the –CH—CH_2_– bond. Its mean plane makes dihedral angles of 7.19 (12) and 71.13 (11)° with those of the thio­phene and toluene rings, respectively. The carbothi­amide group [C(=S)—N] is inclined to the pyrazole ring mean plane by 16.8 (2)°. In the crystal, mol­ecules are linked by N—H⋯S hydrogen bonds, forming chains propagating along [010]. Within the chains, there are N—H⋯π inter­actions present. Between the chains there are weak parallel slipped π–π inter­actions involving inversion-related thio­phene and pyrazole rings [inter-centroid distance = 3.7516 (14) Å; inter-planar distance = 3.5987 (10) Å; slippage = 1.06 Å].

## Abstract 2

In the title compound, the central pyrazole ring adopts a twisted conformation on the –CH—CH_2_– bond and its mean plane makes dihedral angles of 7.19 (12) and 71.13 (11)° with the attached thio­phene and toluene rings, respectively. In the crystal, mol­ecules are linked by N—H⋯S hydrogen bonds, forming chains propagating along [010].

## Chemical context   

Five-membered heterocyclic pyrazole analogues have been used extensively as building blocks in organic synthesis. They have been transformed efficiently into mol­ecules of potential medicinal and pharmaceutical important. Pyrazole derivatives have known to exhibit diverse biological applications such as anti­diabetic,anaesthetic, anti­microbial and anti­oxidant. In addition, they have also shown potential anti­cancer and anti­amoebic activity and to be potent and selective inhibitors of tissue-nonspecific alkaline phosphatase (Sidique *et al.* 2009 ▸). Earlier we synthesized α and β-unsaturated compounds which served as useful inter­mediates for the synthesis of pyrazolines (Manjula *et al.*, 2013 ▸) and thia­zepines (Manjunath *et al.*, 2014 ▸). As part of our ongoing research on pyrazole analogues, the title compound was synthesized and we report herein on its crystal structure. Studies of the biological activity of the title compound are underway and will be reported elsewhere.
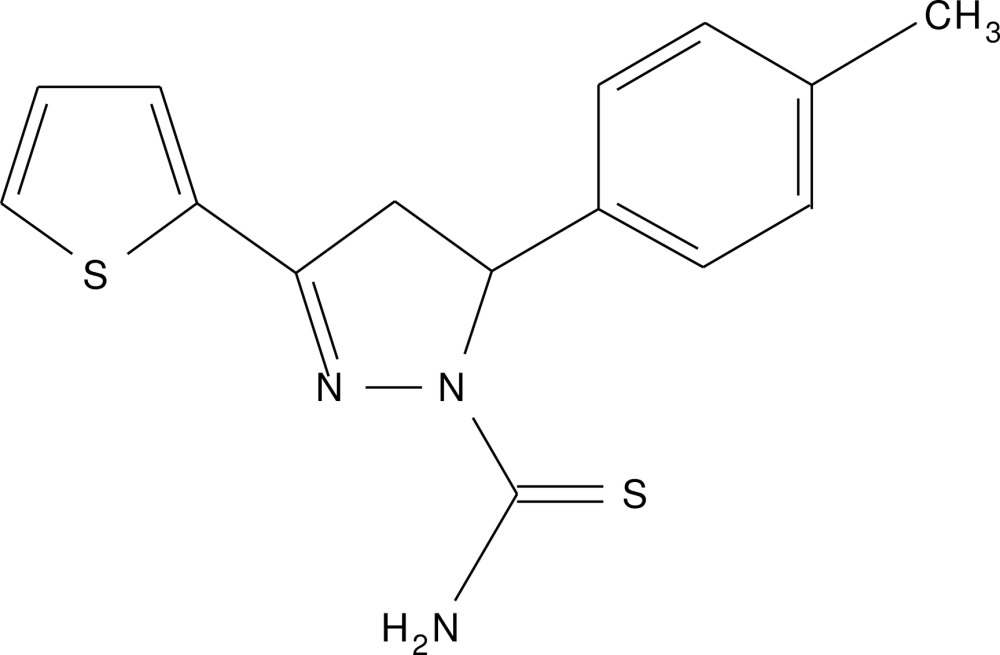


## Structural commentary   

**Figure 1:**
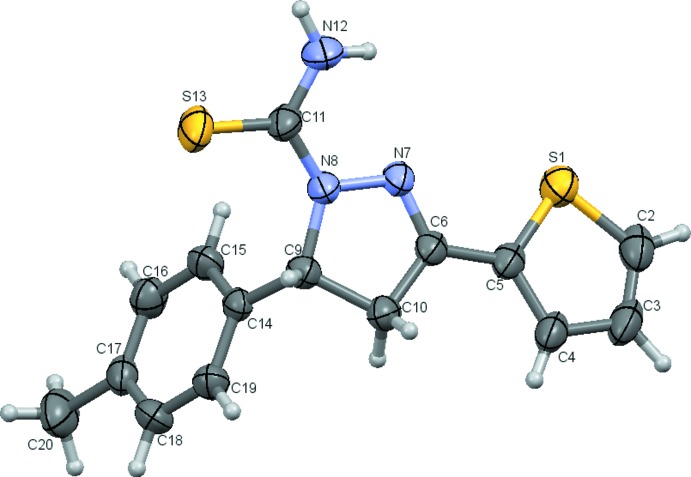
View of the mol­ecular structure of the title compound, showing the atom labelling. Displacement ellipsoids are drawn at the 50% probability level.

The mol­ecular structure of the title compound is illustrated in Fig. 1 ▸. The central pyrazole ring (N7/N8/C8–C10) adopts a twisted conformation with respect to the C9—C10 bond and its mean plane makes dihedral angles of 7.19 (12) and 71.13 (11)° with the thio­phene (S1/C2–C5) and toluene (C14–C19) rings, respectively. The carbothi­amide group [C11(=S13)N12] lies in the plane of the pyrazole ring, as indicated by the torsion angles N12—C11—N8—N7 = 0.6 (3) and S13—C11—N8—N7 = 179.96 (16)°, and adopts +*syn*-periplanar and +*anti*-periplanar conformations, respectively. The title compound possess a chiral center at atom C9 but crystallized as a racemate.

## Supra­molecular features   

**Figure 2:**
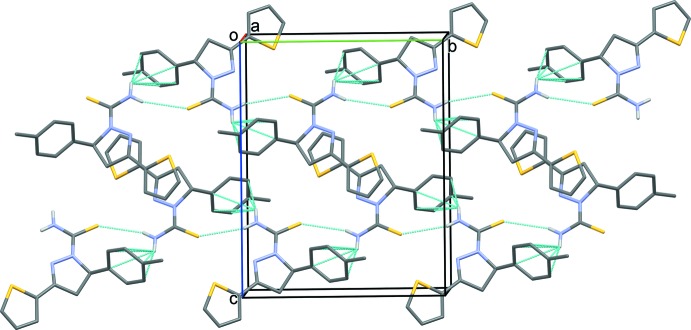
A view along the *a* axis of the crystal packing of the title compound. The hydrogen bonds and C—H⋯π inter­actions are shown as dashed lines (see Table 1 ▸ for details). C-bound H atoms have been omitted for clarity.

In the crystal, mol­ecules are linked by N—H⋯S hydrogen bonds, forming chains propagating along [010]. Within the chains there are N—H⋯π inter­actions involving the toluene ring (Fig. 2 ▸ and Table 1 ▸). Between the chains there are weak parallel slipped π–π inter­actions involving inversion-related thio­phene and pyrazole rings [*Cg*1⋯*Cg*2^i^ = 3.7516 (14) Å; inter-planar distance = 3.5987 (10) Å; slippage = 1.06 Å; *Cg*1 and *Cg*2 are the centroids of rings S1/C2–C5 and N7/N8/C8–C10, respectively; symmetry code: (i) −*x* + 2, −*y* + 1, −*z* + 1].

## Database survey   

A search of the Cambridge Structural Database (Version 5.36, May 2015; Groom & Allen, 2014 ▸) revealed seven structures containing the 3-(thio­phen-2-yl)-pyrazole unit. Amongst these are two thio­amides; the phenyl derivative of the title compound, 5-phenyl-3-(2-thien­yl)-2-pyrazoline-1-thio­amide (HEFXEW; Işık *et al.*, 2006 ▸), and 1-(*N*-ethyl­thio­carbamo­yl)-3,5-bis­(2-thien­yl)-2-pyrazoline (YINFUX; Köysal *et al.*, 2007 ▸). In these two compounds, the pyrazole rings have envelope conformations with the methine C atom as the flap, and the mean planes of the two rings are inclined to one another by 11.98 and 10.13°, respectively. This is in contrast to the situation in the title compound where the pyrazole ring has a twisted conformation on the –CH–CH_2_– bond and its mean plane is inclined to the thio­phene ring by 7.19 (12)°. In the crystal of the phenyl derivative (HEFXEW), mol­ecules are also linked by N—H⋯S hydrogen bonds, forming chains.

## Synthesis and crystallization   

A mixture of 3-(4-methyl­phen­yl)-1-(thio­phen-2-yl)prop-2-en-1-one (0.001 mol) and thio­semicarbazine hydro­chloride (0.01 mol) and potassium hydroxide (0.02 mol) in ethyl alcohol (20 ml) was refluxed on a water bath for 6–8 h. The progress of the reaction was monitored by TLC. After completion of the reaction, the mixture was poured into ice-cold water and stirred. The solid that separated was filtered, and washed with ice-cold water. The product was recrystallized from ethyl alcohol to give the title compound as rectangular yellow crystals. Analysis calculated for C_15_H_15_N_3_S_2_: C, 59.77; H, 5.02; N, 13.94%; found: C, 59.74; H, 5.06; N, 13.88%. ^1^H NMR (CDCl_3_): δ 2.297 (*s*, 3H, CH3), (*dd*, 1H, C4—Hb: *J* = 18.0, 8.5 Hz), (dd, 1H, C4—Hb: *J* 18.0, 8.5 Hz), 5.976–6.013 (*dd*, 1H, C—Ha: *J* = 18.0, 12.0 Hz), 6.163–7.169 (*m*, 7H, Ar—H and thio­phene ring-H), 7.330 (*s*, 2H, –NH_2_). ^13^C NMR (CDCl_3_): δ 43.77, 1 C, C-4), 63.34 (1 C, C-5), 125.35 (2C, Ar—C), 127.88 (1C, 5 m ring-C), 129.57 (1C, Ar—C), 129.67 (1C, Ar—C), 129.72 (1C, 5 m ring-C), 130.01 (1C, 5 m ring-C), 134.12, (1C, 5 m ring-C), 137.31 (1C, Ar—C), 138.67 (1C, Ar—C), 151.38 (1C, C-3), 176.36 (1C, C=S). MS (*m*/*z*): 303 (*M*+2, 10) 302 (*M*+1, 18), 301 (*M*+, 100), 284 (40), 161 (15).

## Refinement   

Crystal data, data collection and structure refinement details are summarized in Table 2 ▸. H atoms were fixed geometrically and allowed to ride on their parent atoms: C—H = 0.93–0.98 Å with *U*_iso_(H) = 1.5*U*_eq_(*C*) for methyl H atoms and 1.2*U*_eq_(C) for other H atoms.

## Supplementary Material

Crystal structure: contains datablock(s) global, I. DOI: 10.1107/S2056989015010774/su5147sup1.cif

Structure factors: contains datablock(s) I. DOI: 10.1107/S2056989015010774/su5147Isup2.hkl

Supporting information file. DOI: 10.1107/S2056989015010774/su5147Isup3.cml

CCDC reference: 1404788

Additional supporting information:  crystallographic information; 3D view; checkCIF report

## Figures and Tables

**Table 1:** Hydrogen-bond geometry (, ) *Cg*3 is the centroid of the benzene ring C14C19.

*D*H*A*	*D*H	H*A*	*D* *A*	*D*H*A*
N12H12*A*S13^i^	0.86	2.83	3.620(2)	154
N12H12*B* *Cg*3^i^	0.86	2.81	3.443(2)	132

Symmetry code: (i) 
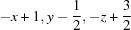
.

**Table 2:** Experimental details

Crystal data	
Chemical formula	C_15_H_15_N_3_S_2_
*M*_r_	301.44
Crystal system, space group	Monoclinic, *P*2_1_/*c*
Temperature (K)	296
*a*, *b*, *c* ()	8.1035(4), 12.0193(5), 15.1312(7)
()	94.347(2)
*V* (^3^)	1469.52(12)
*Z*	4
Radiation type	Cu *K*
(mm^1^)	3.22
Crystal size (mm)	0.27 0.25 0.24

Data collection	
Diffractometer	Bruker X8 Proteum
Absorption correction	Multi-scan (*SADABS*; Bruker, 2013 ▸)
*T*_min_, *T*_max_	0.477, 0.512
No. of measured, independent and observed [*I* > 2(*I*)] reflections	11926, 2397, 2262
*R*_int_	0.044
(sin /)_max_ (^1^)	0.583

Refinement	
*R*[*F* ^2^ > 2(*F* ^2^)], *wR*(*F* ^2^), *S*	0.046, 0.127, 1.07
No. of reflections	2397
No. of parameters	183
H-atom treatment	H-atom parameters constrained
_max_, _min_ (e ^3^)	0.37, 0.44

Computer programs: *APEX2* and *SAINT* (Bruker, 2013 ▸), *SHELXS97* and *SHELXL97* (Sheldrick, 2008 ▸), *Mercury* (Macrae *et al.*, 2008 ▸) and *PLATON* (Spek, 2009 ▸).

## supplementary crystallographic information

### Crystal data

**Table:**

C_15_H_15_N_3_S_2_	*F*(000) = 632
*M**_r_* = 301.44	*D*_x_ = 1.362 Mg m^−^^3^
Monoclinic, *P*2_1_/*c*	Cu *K*α radiation, λ = 1.54178 Å
Hall symbol: -P 2ybc	Cell parameters from 2262 reflections
*a* = 8.1035 (4) Å	θ = 5.5–64.1°
*b* = 12.0193 (5) Å	µ = 3.22 mm^−^^1^
*c* = 15.1312 (7) Å	*T* = 296 K
β = 94.347 (2)°	Rectangle, yellow
*V* = 1469.52 (12) Å^3^	0.27 × 0.25 × 0.24 mm
*Z* = 4	

### Data collection

**Table:**

Bruker X8 Proteum diffractometer	2397 independent reflections
Radiation source: Bruker MicroStar microfocus rotating anode	2262 reflections with *I* > 2σ(*I*)
Graphite monochromator	*R*_int_ = 0.044
Detector resolution: 18.4 pixels mm^-1^	θ_max_ = 64.1°, θ_min_ = 5.5°
φ and ω scans	*h* = −9→9
Absorption correction: multi-scan (*SADABS*; Bruker, 2013)	*k* = −13→13
*T*_min_ = 0.477, *T*_max_ = 0.512	*l* = −16→17
11926 measured reflections	

### Refinement

**Table:**

Refinement on *F*^2^	Secondary atom site location: difference Fourier map
Least-squares matrix: full	Hydrogen site location: inferred from neighbouring sites
*R*[*F*^2^ > 2σ(*F*^2^)] = 0.046	H-atom parameters constrained
*wR*(*F*^2^) = 0.127	*w* = 1/[σ^2^(*F*_o_^2^) + (0.074*P*)^2^ + 0.626*P*] where *P* = (*F*_o_^2^ + 2*F*_c_^2^)/3
*S* = 1.07	(Δ/σ)_max_ < 0.001
2397 reflections	Δρ_max_ = 0.37 e Å^−^^3^
183 parameters	Δρ_min_ = −0.44 e Å^−^^3^
0 restraints	Extinction correction: *SHELXL*, FC^*^=KFC[1+0.001XFC^2^Λ^3^/SIN(2Θ)]^-1/4^
Primary atom site location: structure-invariant direct methods	Extinction coefficient: 0.0160 (12)

### Special details

**Table:**

Geometry. Bond distances, angles *etc*. have been calculated using the rounded fractional coordinates. All su's are estimated from the variances of the (full) variance-covariance matrix. The cell e.s.d.'s are taken into account in the estimation of distances, angles and torsion angles
Refinement. Refinement on *F*^2^ for ALL reflections except those flagged by the user for potential systematic errors. Weighted *R*-factors *wR* and all goodnesses of fit *S* are based on *F*^2^, conventional *R*-factors *R* are based on *F*, with *F* set to zero for negative *F*^2^. The observed criterion of *F*^2^ > σ(*F*^2^) is used only for calculating -*R*-factor-obs *etc*. and is not relevant to the choice of reflections for refinement. *R*-factors based on *F*^2^ are statistically about twice as large as those based on *F*, and *R*-factors based on ALL data will be even larger.

### Fractional atomic coordinates and isotropic or equivalent isotropic displacement parameters (Å^2^)

**Table:**

	*x*	*y*	*z*	*U*_iso_*/*U*_eq_	
S1	0.83886 (8)	0.35843 (5)	0.55113 (4)	0.0527 (2)	
S13	0.41488 (9)	0.76951 (5)	0.77019 (4)	0.0611 (3)	
N7	0.6560 (2)	0.55350 (14)	0.63166 (11)	0.0396 (5)	
N8	0.5837 (2)	0.65224 (14)	0.65865 (11)	0.0418 (6)	
N12	0.5024 (3)	0.55813 (17)	0.77851 (13)	0.0572 (7)	
C2	0.9012 (3)	0.3098 (2)	0.45389 (18)	0.0561 (8)	
C3	0.8661 (3)	0.3801 (2)	0.38623 (17)	0.0613 (9)	
C4	0.7858 (3)	0.4794 (2)	0.41081 (15)	0.0494 (8)	
C5	0.7627 (3)	0.47817 (18)	0.50124 (13)	0.0400 (6)	
C6	0.6841 (2)	0.56438 (17)	0.54966 (13)	0.0368 (6)	
C9	0.5786 (3)	0.73914 (17)	0.58990 (13)	0.0381 (6)	
C10	0.6257 (3)	0.67214 (18)	0.50885 (13)	0.0417 (6)	
C11	0.5039 (3)	0.65429 (18)	0.73411 (14)	0.0438 (7)	
C14	0.6974 (2)	0.83434 (15)	0.61077 (13)	0.0326 (5)	
C15	0.8130 (3)	0.83437 (18)	0.68204 (14)	0.0421 (7)	
C16	0.9258 (3)	0.92083 (19)	0.69439 (15)	0.0469 (7)	
C17	0.9243 (3)	1.00979 (18)	0.63663 (14)	0.0437 (7)	
C18	0.8065 (3)	1.00989 (18)	0.56564 (15)	0.0470 (7)	
C19	0.6951 (3)	0.92360 (18)	0.55243 (14)	0.0419 (6)	
C20	1.0453 (4)	1.1050 (2)	0.65103 (19)	0.0688 (10)	
H2	0.95400	0.24170	0.44810	0.0670*	
H3	0.89180	0.36530	0.32850	0.0740*	
H4	0.75340	0.53690	0.37220	0.0590*	
H9	0.46560	0.76780	0.57940	0.0460*	
H10A	0.53080	0.66110	0.46670	0.0500*	
H10B	0.71300	0.70870	0.47930	0.0500*	
H12A	0.55020	0.50040	0.75860	0.0690*	
H12B	0.45370	0.55400	0.82700	0.0690*	
H15	0.81560	0.77590	0.72240	0.0500*	
H16	1.00410	0.91880	0.74260	0.0560*	
H18	0.80230	1.06920	0.52610	0.0560*	
H19	0.61750	0.92520	0.50390	0.0500*	
H20A	0.98990	1.16790	0.67420	0.1030*	
H20B	1.08790	1.12470	0.59560	0.1030*	
H20C	1.13490	1.08270	0.69240	0.1030*	

### Atomic displacement parameters (Å^2^)

**Table:**

	*U*^11^	*U*^22^	*U*^33^	*U*^12^	*U*^13^	*U*^23^
S1	0.0578 (4)	0.0494 (4)	0.0530 (4)	−0.0001 (3)	0.0172 (3)	−0.0061 (2)
S13	0.0750 (5)	0.0495 (4)	0.0628 (4)	−0.0051 (3)	0.0306 (3)	−0.0180 (3)
N7	0.0483 (10)	0.0317 (9)	0.0408 (9)	−0.0044 (7)	0.0164 (7)	−0.0045 (7)
N8	0.0561 (11)	0.0321 (9)	0.0395 (9)	−0.0044 (8)	0.0188 (8)	−0.0026 (7)
N12	0.0786 (14)	0.0492 (12)	0.0478 (11)	−0.0064 (10)	0.0304 (10)	0.0030 (9)
C2	0.0557 (14)	0.0474 (14)	0.0681 (16)	−0.0092 (11)	0.0240 (12)	−0.0190 (12)
C3	0.0728 (17)	0.0637 (16)	0.0506 (14)	−0.0182 (13)	0.0258 (12)	−0.0257 (13)
C4	0.0583 (14)	0.0489 (13)	0.0429 (12)	−0.0148 (11)	0.0156 (10)	−0.0163 (10)
C5	0.0403 (11)	0.0424 (12)	0.0386 (10)	−0.0145 (9)	0.0112 (8)	−0.0087 (9)
C6	0.0377 (10)	0.0362 (11)	0.0372 (10)	−0.0118 (8)	0.0084 (8)	−0.0055 (8)
C9	0.0409 (11)	0.0348 (11)	0.0391 (11)	−0.0036 (8)	0.0070 (8)	−0.0004 (8)
C10	0.0509 (12)	0.0387 (11)	0.0357 (10)	−0.0121 (9)	0.0050 (9)	−0.0036 (9)
C11	0.0491 (12)	0.0444 (12)	0.0398 (11)	−0.0122 (10)	0.0157 (9)	−0.0080 (9)
C14	0.0362 (10)	0.0275 (9)	0.0349 (9)	0.0015 (8)	0.0087 (8)	−0.0029 (7)
C15	0.0504 (12)	0.0342 (11)	0.0410 (11)	0.0016 (9)	−0.0004 (9)	0.0053 (8)
C16	0.0491 (12)	0.0474 (13)	0.0430 (11)	−0.0019 (10)	−0.0040 (9)	−0.0065 (10)
C17	0.0495 (12)	0.0371 (12)	0.0459 (11)	−0.0077 (9)	0.0137 (9)	−0.0128 (9)
C18	0.0642 (14)	0.0324 (11)	0.0451 (11)	−0.0070 (10)	0.0090 (10)	0.0057 (9)
C19	0.0508 (12)	0.0375 (11)	0.0366 (10)	−0.0002 (9)	−0.0010 (9)	0.0022 (8)
C20	0.0790 (19)	0.0592 (17)	0.0697 (17)	−0.0309 (15)	0.0151 (14)	−0.0182 (14)

### Geometric parameters (Å, º)

**Table:**

S1—C2	1.695 (3)	C15—C16	1.387 (3)
S1—C5	1.718 (2)	C16—C17	1.381 (3)
S13—C11	1.672 (2)	C17—C18	1.382 (3)
N7—N8	1.398 (2)	C17—C20	1.512 (4)
N7—C6	1.285 (3)	C18—C19	1.380 (3)
N8—C9	1.473 (3)	C2—H2	0.9300
N8—C11	1.354 (3)	C3—H3	0.9300
N12—C11	1.337 (3)	C4—H4	0.9300
N12—H12B	0.8600	C9—H9	0.9800
N12—H12A	0.8600	C10—H10A	0.9700
C2—C3	1.341 (4)	C10—H10B	0.9700
C3—C4	1.422 (3)	C15—H15	0.9300
C4—C5	1.395 (3)	C16—H16	0.9300
C5—C6	1.445 (3)	C18—H18	0.9300
C6—C10	1.496 (3)	C19—H19	0.9300
C9—C14	1.513 (3)	C20—H20A	0.9600
C9—C10	1.539 (3)	C20—H20B	0.9600
C14—C19	1.389 (3)	C20—H20C	0.9600
C14—C15	1.374 (3)		
			
C2—S1—C5	91.62 (11)	C18—C17—C20	120.9 (2)
N8—N7—C6	107.73 (16)	C17—C18—C19	121.2 (2)
N7—N8—C9	112.69 (15)	C14—C19—C18	120.8 (2)
N7—N8—C11	119.94 (17)	S1—C2—H2	124.00
C9—N8—C11	126.37 (17)	C3—C2—H2	124.00
H12A—N12—H12B	120.00	C2—C3—H3	123.00
C11—N12—H12A	120.00	C4—C3—H3	123.00
C11—N12—H12B	120.00	C3—C4—H4	125.00
S1—C2—C3	112.67 (19)	C5—C4—H4	125.00
C2—C3—C4	113.8 (2)	N8—C9—H9	110.00
C3—C4—C5	110.2 (2)	C10—C9—H9	110.00
S1—C5—C4	111.68 (17)	C14—C9—H9	110.00
S1—C5—C6	122.38 (15)	C6—C10—H10A	111.00
C4—C5—C6	125.9 (2)	C6—C10—H10B	111.00
N7—C6—C10	114.39 (17)	C9—C10—H10A	111.00
N7—C6—C5	122.27 (18)	C9—C10—H10B	111.00
C5—C6—C10	123.33 (17)	H10A—C10—H10B	109.00
N8—C9—C10	101.33 (16)	C14—C15—H15	120.00
N8—C9—C14	113.93 (17)	C16—C15—H15	120.00
C10—C9—C14	111.69 (18)	C15—C16—H16	119.00
C6—C10—C9	102.35 (16)	C17—C16—H16	119.00
N8—C11—N12	115.5 (2)	C17—C18—H18	119.00
S13—C11—N12	122.08 (18)	C19—C18—H18	119.00
S13—C11—N8	122.39 (16)	C14—C19—H19	120.00
C9—C14—C15	123.31 (18)	C18—C19—H19	120.00
C9—C14—C19	118.29 (18)	C17—C20—H20A	109.00
C15—C14—C19	118.32 (18)	C17—C20—H20B	109.00
C14—C15—C16	120.6 (2)	C17—C20—H20C	109.00
C15—C16—C17	121.4 (2)	H20A—C20—H20B	110.00
C16—C17—C20	121.4 (2)	H20A—C20—H20C	109.00
C16—C17—C18	117.7 (2)	H20B—C20—H20C	110.00
			
C5—S1—C2—C3	−0.2 (2)	C4—C5—C6—N7	174.6 (2)
C2—S1—C5—C4	0.1 (2)	C4—C5—C6—C10	−4.2 (3)
C2—S1—C5—C6	179.6 (2)	C5—C6—C10—C9	−171.56 (19)
C6—N7—N8—C9	−5.9 (2)	N7—C6—C10—C9	9.6 (2)
C6—N7—N8—C11	163.41 (18)	C10—C9—C14—C15	−107.0 (2)
N8—N7—C6—C5	178.34 (17)	C10—C9—C14—C19	69.6 (2)
N8—N7—C6—C10	−2.8 (2)	N8—C9—C14—C19	−176.34 (18)
C11—N8—C9—C14	82.7 (3)	N8—C9—C10—C6	−11.5 (2)
C9—N8—C11—N12	168.3 (2)	C14—C9—C10—C6	110.18 (18)
N7—N8—C11—S13	179.96 (16)	N8—C9—C14—C15	7.1 (3)
N7—N8—C9—C10	11.3 (2)	C9—C14—C15—C16	175.7 (2)
C11—N8—C9—C10	−157.2 (2)	C19—C14—C15—C16	−0.9 (3)
N7—N8—C11—N12	0.6 (3)	C9—C14—C19—C18	−176.6 (2)
N7—N8—C9—C14	−108.84 (18)	C15—C14—C19—C18	0.2 (3)
C9—N8—C11—S13	−12.4 (3)	C14—C15—C16—C17	0.9 (4)
S1—C2—C3—C4	0.3 (3)	C15—C16—C17—C18	−0.1 (4)
C2—C3—C4—C5	−0.3 (3)	C15—C16—C17—C20	178.9 (2)
C3—C4—C5—S1	0.1 (3)	C16—C17—C18—C19	−0.6 (3)
C3—C4—C5—C6	−179.4 (2)	C20—C17—C18—C19	−179.6 (2)
S1—C5—C6—N7	−4.9 (3)	C17—C18—C19—C14	0.6 (3)
S1—C5—C6—C10	176.33 (17)		

### Hydrogen-bond geometry (Å, º)

Cg3 is the centroid of the benzene ring C14–C19.

**Table:**

*D*—H···*A*	*D*—H	H···*A*	*D*···*A*	*D*—H···*A*
N12—H12*A*···S13^i^	0.86	2.83	3.620 (2)	154
N12—H12*B*···*Cg*3^i^	0.86	2.81	3.443 (2)	132

Symmetry code: (i) −*x*+1, *y*−1/2, −*z*+3/2.
